# Differences in Children’s Social Development: How Migration Background Impacts the Effect of Early Institutional Childcare Upon Children’s Prosocial Behavior and Peer Problems

**DOI:** 10.3389/fpsyg.2021.614844

**Published:** 2021-02-16

**Authors:** Kira Konrad-Ristau, Lars Burghardt

**Affiliations:** ^1^Psychology I – Developmental Psychology, University of Bamberg, Bamberg, Germany; ^2^Chair of Early Childhood Education, University of Bamberg, Bamberg, Germany

**Keywords:** migration background, early childhood education and care (ECEC), children’s social development, disadvantaged children, prosocial behavior, peer problems, childcare

## Abstract

This article focuses on the early years of children from immigrant families in Germany. Research has documented disparities in young children’s development correlating with their family background (e.g., immigrant or ethnic minority status), making clear the importance of early intervention. Institutional childcare—as an early intervention for children at risk—plays an important role in Germany, as 34.3% of children below the age of three and 93% of children above that age are in external childcare. This paper focuses on the extent to which children from families with a background of migration differ in their social development when considering their age of entry into early external childcare (and thus its duration). Data from the infant cohort study of the German National Educational Panel Study (NEPS, *N* = 1,846) is used to analyze the impact of early institutional childcare before the age of 3 years on children’s social competence at the age of 5 years, controlling for gender, siblings, temperament, home learning activities, and socioeconomic status. Results show the effects of duration of early external childcare on peer problems for children from families with a background of migration, in such a way that children who attend early external childcare for more than 1 year before the age of three show less problem behavior with peers than those who attend for less than a year. These findings have equity implications for children with a migration background living in Germany, especially as the proportion of these children is trending upwards.

## Introduction

The effects of social disparities on child development have been extensively analyzed and discussed, and the topic is assigned high theoretical and social relevant (Becker, 2011; Kalb, 2017). The main points of interest are how these disparities arise, how they persist, and how they can be reduced. Disparities in child development emerge early in childhood with significant consequences for children’s further development and educational outcomes (Hart and Risley, 1995; Cunha and Heckman, 2007; Becker and Reimer, 2010; Cunha et al., 2010). Children who have an increased developmental risk due to various stresses, such as a lower educational status or a migration background^1^, are referred to as “children at risk” in the applicable early childhood education plans (e.g., that of the German federal state of Bavaria; Staatsinstitut für Frühpädagogik, 2007). Early interventions are designed to support children at risk and mitigate early social inequalities in order to contribute to the creation of equal opportunities for all children, regardless of their origin. Early institutional childcare is considered as one form of early intervention and has been shown to positively impact children’s development (Melhuish et al., 2015). In fact, education starts long before school entry (Belsky et al., 2007; Weinert et al., 2016, 2017; Kalb, 2017). In Germany, children’s legal rights regarding access to early external childcare have changed in recent years, leading to an increased need (as well as a new opportunity) to evaluate the impact of early institutional childcare, especially on children at risk. Since 1996, every child aged 3 years or older has been entitled to a place in a German childcare center. In 2013, this right was expanded: Since then, each child older than 1 year has been entitled to a place in a childcare center or in family-based daycare. Whereas in 2008 17.6% of children under the age of three attended childcare in Germany, the proportion has more than doubled in the last 10 years, rising to 34.3% in 2019 (0–1-year-olds: 1.9%, 1–2-year-olds: 37.1%, 2–3-year-olds: 63.2%; Statistisches Bundesamt, 2019). The overall demand for childcare for children under the age of three is considerably higher than available places, trending upwards (Alt et al., 2014). Looking at the users of external childcare, we see differences with regard to the structural characteristics of the families: Parents with higher socioeconomic status (SES) are more likely to use early external childcare services (in the first 3 years of a child’s life), while parents of children with a migration background are less likely to (for an overview: Burghardt, 2019a). Although international findings are inconsistent and reveal that not in all countries children with a migration background enter external childcare at a later time (Sylva et al., 2007), there is a clear trend in German research. Studies show that children with a migration background are less likely to attend early childcare in Germany (Eckhardt and Riedel, 2012); when they do attend, they start later (Burghardt, 2018). Of all infants who received childcare in their first 3 years of life, only every fifth child has a migration background, even though 40.6% of all children under the age of five have this familial status (Statistisches Bundesamt, 2018; Statistische Ämter des Bundes und der Länder, 2019). The fact that children with a migration background and those from families with a low socioeconomic status are comparatively less likely to attend early childhood institutions emphasizes that inequalities already exist at this young age (Becker, 2012). In this context, it is notable that research in Germany indicates a significant correlation between the families’ socioeconomic status and the migration background of a family (von Marées and Petermann, 2010). Consequently, the question arises as to the extent to which particularly children at risk may benefit from early institutional childcare. Research (Anders, 2013; Melhuish et al., 2015) has documented that attending an early childhood out-of-home facility impacts on the development of children. It is explicitly and implicitly assumed that the effects of stimulation in early educational institutions are particularly evident among children from families with a migration background, a low socioeconomic status, and a low quality and/or quantity of stimulation at home (Roßbach et al., 2008). Theory as well as research proposes a widely-held expectation: institutional childcare may reduce the effects of low stimulation quality in the home environment and is therefore especially important for children at risk (NICHD Early Child Care Research Network, 2000; Watamura et al., 2011). As the fact that children with a migration background are less likely to attend early institutional childcare in Germany and when they enter, they are comparatively older, it raises the question if an earlier start would be especially beneficial for those children. The analysis of this question has practical and political implications, as there has been a debate in Germany about possible barriers to access institutional childcare for disadvantaged groups, such as families with a migration background (Berg-Lupper, 2006; Lokhande, 2013; Burghardt, 2019b). Current research reinforces the assumption of access barriers as they show that this group of parents is just as likely to try to access childcare (Alt et al., 2016), but they are often unable to realize their aspirations. The authors describe those parents as “non-voluntary non-users” (p. 702).

Research on the effects of institutional childcare differs in the specific features of childcare that are analyzed, mainly quality, duration and intensity (for an overwiew: Melhuish et al., 2015; Holl et al., 2020). It is important to investigate the specific features in the contexts of the countries’ early childhood education and care systems, as they vary (OECD, 2013). For example, the study of the NICHD Early Child Care Research Network (2003) reports negative effects of institutional childcare for children who experience a high amount of hours of childcare a week. They also find quite a significant amount of children who are cared for in their first year of life. In Germany, we hardly find such high numbers of intensity and less than 2% of children under the age of 1 year are in institutional childcare (Statistisches Bundesamt, 2019). As described in our rationale above, our focus lies on the effect of duration as the results may have specific implications for the suppliers of external childcare and policy makers. The limitations of our analysis with regard to other childcare features like quality are addressed in section “Discussion.”

While positive effects on children’s cognitive and linguistic development have been documented across many studies (Becker, 2006, 2011; Belsky et al., 2007; Cunha and Heckman, 2007), this does not hold to the same extent for social facets of development, especially for children under the age of three. Broadly speaking, social development reflects all changes in relationships or in the social environment over time (Schmidt-Denter, 2005). Children’s repertory of social behavior with adults and peers expands and diversifies enormously during their first years of life. Children acquire skills that enable them, among other things, to initiate and maintain social interactions and friendships, to cooperate in play situations and to solve conflicts, to understand and negotiate rules, and to express affection and demarcation appropriately (Viernickel, 2013). Since the development of competencies is the result of the interplay of multiple experiences, both the family situation and early institutional childcare—in addition to the characteristics of the child—play a decisive role in the social development of children (Bronfenbrenner and Morris, 2006). The structural characteristics of the family, such as socioeconomic and educational status (including income and home learning environment), but also external surroundings such as institutional childcare, account for variability in infants’ social competence (Bronfenbrenner and Morris, 2006; Halle et al., 2009; Blomeyer et al., 2010; Kalb, 2017; Linberg A., 2017; Rose et al., 2018). Particularly for social competencies, such as problem behavior with peers and prosocial behavior, early external childcare seems to be highly relevant, due to the resulting increase in (and in some cases even first) interactions with peers and adults in the absence of a child’s parents (Pfeffer, 2017; Linberg A., 2017; Linberg et al., 2019). Family context variables, such as low income, lack of social support, and migration background, are considered to be social risk factors which are potentially associated with children’s development (Foster et al., 2005; Voltmer and von Salisch, 2019). In particular, children who grow up under conditions of social risk are already disadvantaged in their development at an early age compared to other children, as shown e.g., by research results on children under the age of 3 years obtained using data from the German National Educational Panel Study (Weinert et al., 2017). Yet not only the family and the institutional childcare environment are relevant for a child’s social development, but also the characteristics of the child him- or herself, e.g., his/her temperament. Linberg et al. (2019) note that children with a difficult temperament tend to show less prosocial behavior when they are not enrolled in a childcare setting. Children with a mid-range difficult temperament seem to benefit especially from institutional childcare, as they show the highest scores for prosocial behavior when enrolled in early institutional childcare for two to 3 years.

The present article focuses on the impact of external childcare experiences before the age of three on children’s later social development in Germany. Of particular interest is whether children with a background of migration can benefit from an early start in childcare, as they tend to start using institutional childcare later.

## Social Disparities in Early Childhood

The effects of social disparities can already be observed in early childhood, and the relevant mechanisms discussed in the literature are complex (Linberg T., 2017). In particular, it has been suggested that early institutional childcare is especially beneficial for disadvantaged children. Melhuish et al. (2015) emphasize the importance of high quality early external childcare for disadvantaged children: “High-quality childcare has been associated with benefits for children’s development, with the strongest effects for children from disadvantaged backgrounds” (p. 81). Both external childcare and the family a child grows up in are important learning environments for explaining developmental progress, as the bio-ecological model proposed by Bronfenbrenner and Morris (2006) implies. Further, early developmental disparities are observed in both cognitive and social development (Cunha and Heckman, 2007; Cunha et al., 2010).

## Early External Childcare and Social Development

Yet, to date, the empirical results concerning the effects of early external childcare on social development are inconsistent, which is partly due to the focus of the studies as they address different features of childcare like quality, duration or intensity. This gets even more complex as we find studies who only address one of the features or combine them in their analysis. In the course of the American NICHD study, those children who spent more time in early institutional childcare showed comparatively less prosocial and more problem behavior with peers at the age of four, especially when the children attended early institutional childcare in their first 6 months of life (NICHD Early Child Care Research Network, 2003). Other results indicate no effect of the age of entry into early external childcare on social development (Dearing et al., 2015), at least under certain conditions (moderate amount of hours of external childcare) or even positive effects of time spent in early institutional childcare for children speaking a minority language (Houng et al., 2011). Additionally, a Swedish study finds evidence that the timing of entry into early institutional childcare affects children’s social behavior. Children who attended institutional childcare before the age of two (at least 25 h/week) were rated as more social by their teachers than children who received care outside the family at a later age or not at all (Andersson, 1989). A recent systematic review reveals that a positive effect of early institutional childcare on social behavior was found in all studies analyzed (Holl et al., 2020). Within their review, Holl et al. (2020) conclude a clear direction of the results that institutional childcare has rather a positive than a negative impact on children’s social well-being. Children who attended institutional childcare were rated as more socially competent by their parents, and fewer behavioral problems were observed among them than among children without outside care. Importantly, study results cannot be interpreted universally, since childcare systems differ substantially both in their underlying organizational conception and with regard to levels of actual use and demand; also highly relevant are a nation’s specific parental leave regulations (Waldfogel, 2001; International Labour Office, 2013; OECD, 2013). Another possible reason for the inconsistency of research results is the application of different approaches and designs in the studies. With respect to Germany, the NUBBEK study, a representative cross-sectional large scale study, documents that children exhibited fewer peer problems at the age of two and four if they spent more time in early external childcare before the age of three (Tietze et al., 2013). Another German study drawing on NEPS data reveals that children who attend institutional childcare for a longer period before the age of three demonstrated less problem behavior with peers than children without external childcare experience (Linberg et al., 2019). Contrary to the NICHD study, Linberg et al. (2019) do not find any negative effects of early external childcare on later prosocial behavior, but actually a slight positive drift while using a set of control variables to control for potential selection effects into early institutional childcare like SES, income, or migration background. The results were stable even when considering intensity of care, group size, and activities in childcare institutions. There is a difficulty relating the results of various studies to each other as—depending on the assessment instrument used—social competence is often conceptualized and recorded rather differently (Viernickel, 2013). In the following, we present the rationale for the conceptualization of social development in this paper. With respect to children’s social development, external childcare is of special significance due to the increase in interactions with peers; it is the first learning environment besides the family where infants are in contact with other children for a substantial amount of time (Brownell, 2008; Pfeffer, 2017). Children with a migration background face a double transition during this first contact with peers and other adults outside their own family. On one hand, like all children, they have to handle a more varied, unfamiliar environment, very different from their home lives; on the other they are participating in a context which is also new from a cultural and linguistic point of view (Picchio and Mayer, 2019). However, while the experiences children make in early institutional childcare might be stimulating for their prosocial behavior, they could also be stressful with respect to conflicts with peers due to their still limited abilities to solve them (Howes, 1987). Thus, experiences in early childcare may be crucial for social development (Goodman, 2016) and can thus, above all, be associated both with children’s problem behavior with peers and their prosocial behavior, which is encouraged in new childcare group situations.

## The Present Study

However, when considering children at risk, especially children with a migration background, there is insufficient research, especially compared to studies focusing on the general effects of early external childcare on children’s social development. Felfe and Lalive (2012) note that infants, boys, and children from families with lower socioeconomic status tend to benefit more in terms of child development. This is especially relevant since families with a migration background often also have a lower socioeconomic status, as noted earlier (von Marées and Petermann, 2010). Another study establishes that children with a migration background who have experienced external childcare exhibit higher overall levels of wellbeing (Kaiser and Bauer, 2019). Children with a migration background benefit particularly from attending an external childcare center for longer (i.e., an earlier entry into external childcare), especially when research focuses on school readiness (Biedinger et al., 2007). Much research concerning children with a migration background focuses on the linguistic development of those children and whether an earlier entry into (i.e., longer duration of) external childcare reduces their poorer language skills in the majority language (Paetsch et al., 2014; Klein and Sonntag, 2017). These studies often concentrate on the gap between children with and without a migration background when starting school (Currie and Thomas, 1996; Magnuson et al., 2006; Becker, 2016).

To summarize, the external childcare environment—in addition to the home learning environment and child characteristics—contributes to child development. However, the level of interaction between social disparities and external childcare is still the subject of some debate. Especially the question as to whether children with a migration background as an underrepresented participant group in research benefit specifically from early external childcare is of significant political and scientific relevance. Thus, the present study aims to provide an analysis of group effects of different durations of early external childcare (in the first 3 years of life) in Germany and later social competence at the age of five.

## Research Question

In what way is the duration of early external childcare (before the age of three) associated with later social competence and problem behavior with peers for 5-year-old children with a migration background?

The focus lies on whether, and to what extent, children with a migration background differ in their social development from one another depending on the time they spend in early external childcare. The intention is to investigate whether there are group differences in peer problems and prosocial behavior at the age of five according to the time spent in early external childcare for children with a migration background. To draw a complete picture and to control for potential selection effects in early external childcare, the following control variables need to be included in the analyses, as these variables might influence differences in social development: the child’s gender, number of siblings, the child’s temperament, home learning activities as an indicator for stimulation at home, and the socioeconomic status of the family. Including gender is relevant, as social development differs by gender, and research has shown that spending more time in early institutional childcare might have different effects depending on the child’s gender (Arace et al., 2019). It also appears that having siblings is significantly related, on the one hand, to later social competencies, such as forming better relationships and learning to resolve conflicts (Brody, 2004; Downey et al., 2013), and also, on the other, to a reduced likelihood of attending institutional childcare (Burghardt, 2019a). Integrating the child’s temperament into the analysis is of particular significance because studies focusing on social development have shown that both significant effects on social development and differential effects of childcare depend on the child’s temperament (Crockenberg, 2003; Arace et al., 2019). Additionally, aspects of the family a child lives in have to be taken into account: a stimulating home environment has been shown to predict children’s social development (Rose et al., 2018). Home learning activities serve as an indicator of stimulation at home that support children’s development (Lehrl et al., 2020) and are often described as the prime engine in child development. Another key variable, when analyzing children’s social development, are socioeconomic family factors. Research has documented interrelations between children’s social development and family factors such as income, or the mother’s educational level (Hartas, 2013). By including these variables in our analyses, we try to do justice to the complexity found in the theoretical model developed by Bronfenbrenner and Morris (2006) and in the state of research in general, and to control for relevant variables affecting social development and selection into institutional childcare.

## Materials and Methods

### Data

The infant cohort study of the German National Education Panel Study^2^ (NEPS; Blossfeld and Roßbach(eds), 2019) forms the data basis for our analyses. The target population of the infant cohort was defined as all children born in Germany from February to July 2012. At the start of the survey the children had to be at least 6 months old, but no older than 8 months. The access to the families was granted via a register-based sample of addresses available at municipal level. The sampling model involved a two-stage disproportional random selection stratified by municipality size classes, with the first sampling stage comprising the municipalities and the second comprising the target population of children born from February to July 2012. A total of 8,483 addresses were randomly selected (Attig et al., 2014). In this way a representative sample of 3,481 children (and their families) who were about 7 months old at the time of the first survey and were reassessed at about 17 months (wave 2), 26 months (wave 3), 38 months (wave 4), 48 months (wave 5), and 60 months of age (wave 6) was gathered. Over time, the cohort reduced in size, resulting in 2,209 children and their families in wave six.

### Sample

The present study uses data on social development from wave six when the children were between 57 and 66 months old (*M* = 5.11 years). The data necessary to carry out the analyses comprised variables on migration background, the duration of the children’s attendance in early institutional childcare, and an assessment of the children’s social development. An initial descriptive examination of the data reduces the sixth wave to 454 children with a migration background (230 girls; 50.7% and 224 boys; 49.3%) and 1,392 children without a migration background (694 girls; 49.9% and 698 boys; 50.1%). In other words, 24.6% of our sample can be identified as children with a migration background. Migration background is assessed via the parents’ first spoken language at home (0 = no migration background, 1 = migration background). The migration background is coded as 1 if one of the parents has not acquired German as a first language. The minority first languages of the parents in the present sample are mainly Russian, Polish, and Turkish, but 17 other first languages are also represented in the sample. In our sample, in 97.14% of the families the mother is the respondent during the survey (99.57% for the children without a migration background). When the survey started, 80.40% of the respondents were married (72.05% for the children without a migration background). In addition, 44.48% of the respondents among families with a migration background have a university degree, compared to 65.52% among families without a migration background.

### Early External Childcare

The duration of early institutional childcare represents how many months an infant has already attended external childcare before reaching the age of 3 years (wave 4 of NEPS—starting cohort I; 1 = up to 1 year, 2 = 1–2 years, 3 = over 2 years). Looking at the numbers of children attending early external childcare in Germany, it becomes clear that a division into three groups is reasonable since there are drastic increases in the attendance rates (0–1-year-olds: 1.9%, 1–2-year-olds: 37.1%, 2–3-year-olds: 63.2%; Statistisches Bundesamt, 2019). Thus, most children in Germany have only 1–2 years of experience with early institutional childcare. This is also related to the existing parental leave laws in Germany, which replace the income loss by 65% for a maximum of 14 months and since 2015 even allow another 4 months of combining parental allowance and part-time work (Federal Ministry for Family Affairs, Senior Citizens, Women and Youth, 2015). However, since children with a migration background enter childcare at a later age (Burghardt, 2018) and are an underrepresented participant group in research, it seems interesting to ask whether their earlier entry into childcare is a factor in their social development.

### Social Development

Social development is measured by using the *Strength and Difficulties Questionnaire* (SDQ; Goodman, 2016). This instrument measures behavioral strengths and difficulties in children and adolescents aged between 4 and 16 years. The questionnaire, which is filled in by a parent, consists of 25 items on emotional problems, behavioral problems, hyperactivity, problem behavior with peers, and prosocial behavior. In the wording attention was paid to a balance of positive and negative aspects of children’s behavior (Viernickel, 2013; Goodman, 2016). We included the scales *problem behavior with peers* and *prosocial behavior* in our paper as they both are highly relevant in the context of early institutional childcare (Goodman, 2016). They are each composed of five items with a three-level answer scale (0 = not applicable, 1 = partially applicable, 2 = clearly applicable). The peer problem scale (Cronbach’s α = 0.58) includes items such as “Gets along better with adults than with other children” or “Is teased or bullied by others” and is used as an indicator for behavioral problems. In contrast, the scale for prosocial behavior (Cronbach’s α = 0.68) is measured using items such as “Loves younger children” or “Willingness to help when others are hurt, sick, or sad.” Due to the low Cronbach’s α, the model was tested for fit as a two-factor and single-factor model. Since the two-factor model shows better model fits than the single factor model, we continued working with the two-factor solution (model fits in the Supplementary Material). For problem behavior with peers, positively phrased items (in the case of prosocial behavior, negatively phrased items) are recoded and then calculated two indicators by using the mean, one for peer problems and one for prosocial behavior. High scores on the prosocial subscale reflect the child’s strengths, whereas high scores on the problem behavior scale reflect social difficulties with peers (Woerner et al., 2002; Goodman, 2016).

### Control Variables

The following control variables are included in the analyses: the child’s gender (0 = male, 1 = female), number of siblings, the socioeconomic status of the family, home learning activities, and the child’s temperament. The socioeconomic status of the family is measured by the Highest International Socioeconomic Index (HISEI, Ganzeboom et al., 1992) in the family. To control for the home environment, we computed an average score using all available items concerning the frequency of joint activities (0 = never; 7 = several times a day) to promote child development at home (e.g., reading aloud, painting, etc.) from waves 3–5 of the NEPS (Cronbach’s α = 0.79). Three scales in NEPS represent the child’s temperament, each including three items from the Children’s Behavior Questionnaire (CBQ, waves 4–6) assessed on a seven-point Likert scale (0 = does not apply at all; 7 = applies completely): negative affect (Cronbach’s α = 0.69), surgency (Cronbach’s α = 0.61), and effortful control (Cronbach’s α = 0.40) (Rothbart et al., 2001). The scale for negative affect includes items such as “Is very hard to calm down when she is excited.” The scale for surgency consists of items such as “Is full of energy, even in the evening” and the scale for effortful control incorporates items like “Likes calming rhythmic activities such as rocking or swaying.” Indicators for each scale are averaged across items and waves.

### Analysis Plan

All statistical analyses are carried out with the Statistical Program SPSS Version 25 (IBM Corp, 2017). We first report relevant descriptives and bivariate correlations for all relevant study variables. To analyze the effects of the duration of early external childcare on the social behavior of children with a migration background, a multivariate analysis of variance is performed including all control variables. To compare the results, an additional multivariate analysis of variance for the children without a migration background is computed. In terms of the homogeneity of the groups’ error variances for both peer problems and prosocial behavior, the requirements do not necessarily have to be met due to the large sample size; an analysis of variance is considered robust in such a case (Lüpsen, 2019). For this reason, the results of the variance analyses can be considered valid.

### Dealing With Missing Values

As described earlier, requirements for the analyses include information about migration background, the duration of a child’s attendance of early institutional childcare, and the SDQ estimates of the children’s peer problems and prosocial behavior. This condition results in a reduced sample size of 454 children with a migration background (82% of the total available number of children with a migration background in wave six of the newborn cohort) and 1,392 children without a migration background (84% of the total available number in wave six of the newborn cohort). For the variables used in the model, the missing values of the reduced sample of 454 children with a migration background still range from 0% (gender, siblings, HISEI) to 15.2% (home learning activities). A comparison of the mean values (duration of childcare, problem behavior with peers, and prosocial behavior) of the initial sample (454 children) with the mean values of the reduced sample (379 children) reveals no significant differences and therefore no implantation was performed, as different results are not expected. The same applies to the due to missings reduced sample (1,226 children) of the children without a migration background which serves as the control group in the present study. The exact mean values are reported in Supplementary Material.

## Results

### Descriptive Results

Descriptives of all included variables for children with a migration background are shown in Table 1. The mean of problem behavior with peers is 1.38 (*SD* = 1.39) and of prosocial behavior it is 8.07 (*SD* = 1.68). The duration of early external childcare for children with a migration background ranges between 0 and 34 months (*M* = 19.14, *SD* = 10.46). The mean of the highest socioeconomic status of the families is 58.99 (*SD* = 20.57) and the gender ratio is balanced.

**TABLE 1:** Descriptive data on variables in the analyses (children with a migration background).

	*N*	Mean	*SD*	Minimum	Maximum
**Dependent variables**					
Problem behavior with peers (0 = low, 10 = high)	454	1.38	1.39	0	6
Prosocial behavior (0 = low, 10 = high)	454	8.07	1.68	2	10
**Control variables**					
Child’s gender (0 = male, 1 = female)	454	0.51	0.50	0	1
Number of siblings	454	1.05	1.06	0	7
Temperament: negative affect (0 = easy, 6 = difficult temperament)	453	3.35	1.20	0	6
Temperament: surgency (0 = easy, 6 = difficult temperament)	449	4.39	1.04	0.33	6
Temperament: effortful control (0 = easy, 6 = difficult temperament)	451	1.60	1.04	0	5.33
Home learning environment (HLE; 0 = low, 7 = high)	385	4.37	0.80	0.94	6.15
Family’s highest socioeconomic status (HISEI)	454	58.99	20.57	14.21	88.96
**Independent variable**					
Duration in childcare before age 3 (in months)	454	19.14	10.46	0	34

To increase the comparability we also included descriptives of all included variables for children without a migration background in Table 2. The mean of problem behavior with peers is 0.97 (*SD* = 1.30) and of prosocial behavior it is 7.92 (*SD* = 1.55). The duration of early institutional childcare for those children ranges between 0 and 35 months (*M* = 22.24, *SD* = 8.20). The mean of the highest socioeconomic status of the families is 68.24 (*SD* = 16.04).

**TABLE 2:** Descriptive data on variables in the analyses (children without a migration background).

	*N*	Mean	*SD*	Minimum	Maximum
**Dependent variables**					
Problem behavior with peers (0 = low, 10 = high)	1,392	0.97	1.30	0	8
Prosocial behavior (0 = low, 10 = high)	1,392	7.92	1.55	1	10
**Control variables**					
Child’s gender (0 = male, 1 = female)	1,392	0.50	0.50	0	1
Number of siblings	1,392	0.97	0.86	0	8
Temperament: negative affect (0 = easy, 6 = difficult temperament)	1,389	3.41	1.05	0	6
Temperament: surgency (0 = easy, 6 = difficult temperament)	1,389	4.20	1.01	0.33	6
Temperament: effortful control (0 = easy, 6 = difficult temperament)	1,387	1.52	0.90	0	5.33
Home learning environment (HLE; 0 = low, 7 = high)	1,236	4.36	0.73	1.79	6.36
Family’s highest socioeconomic status (HISEI)	1,392	68.24	16.04	16.36	88.96
**Independent variable**					
Duration in childcare before age 3 (in months)	1,392	22.24	8.20	0	35

In order to give readers a first impression of the data, the SDQ results of the sample of children with a migration background are displayed in Table 3. The children are classified into the categories “typical,” “borderline,” and “conspicuous.” For the German version of the SDQ, Woerner et al. (2002) select the categories in such a way that approximately 80% of children are classified as “typical,” 10% as ‘borderline’ and 10% as “conspicuous.” Levels of problem behavior with peers (*M* = 1.38, *SD* = 1.39) and prosocial behavior (*M* = 8.07, *SD* = 1.68) are established as mostly inconspicuous in the present sample.

**TABLE 3:** Classification of the children with a migration background via the SDQ scales.

	SDQ distribution					
	
	*n*	Normal	*n*	Borderline	*n*	Conspicuous
Problem behavior with peers	412	90.8%	40	8.8%	2	0.4%
Prosocial behavior	420	92.5%	18	4.0%	16	3.5%

**N = 454.**

A more detailed analysis of the descriptive results when differentiating them for duration of childcare reveals small differences in the mean values of the individual groups with regard to social competence. Table 4 presents the means and standard deviations divided by groups (duration: under 1 year, 1–2 years, over 2 years) for children with a migration background. The descriptive analysis shows lower numerical values in the children’s problem behavior with peers if they attended early external childcare for longer before the age of 3 years, and similar values independent of childcare duration in their prosocial behavior.

**TABLE 4:** Means and standard deviations for peer problems and prosocial behavior of children with a migration background.

	Duration					
	
	Below 1 year		1–2 years		Above 2 years	
			
	*n*	*M* (*SD*)	*n*	*M* (*SD*)	*n*	*M* (*SD*)
Problem behavior with peers	116	1.74 (1.57)	141	1.30 (1.34)	197	1.23 (1.28)
Prosocial behavior	116	8.02 (1.76)	141	8.23 (1.63)	197	7.98 (1.66)

**N = 454.**

### Correlations

Table 5 provides an overview of the correlations between the social competencies, the duration of early external childcare, and the following control variables: gender; siblings; the CBQ scales negative affect, surgency, and effortful control; home learning activities; and HISEI. We find a correlation for children with a migration background between problem behavior with peers and the variables siblings, negative affect (CBQ), HISEI, and the duration of early external childcare (*r* = −0.19^∗∗^ to *r* = 0.19^∗∗^, ^∗^*p* < 0.05, ^∗∗^*p* < 0.01). Additionally, the variables gender, negative affect (CBQ), effortful control (CBQ), and home learning activities correlate with prosocial behavior (*r* = −0.23^∗∗^ to *r* = 0.19^∗∗^, ^∗^*p* < 0.05,^∗∗^*p* < 0.01). There is also a correlation between peer problems and prosocial behavior (*r* = −0.15, *p* < 0.01). The correlation analysis shows that girls are more likely to be assessed as inconspicuous in terms of their prosocial behavior than boys. Further, a child’s temperament plays a role in relation to both peer problems and prosocial behavior. The number of siblings is positively related to children’s problem behavior with peers, and home learning activities show a positive effect on prosocial behavior. Furthermore, the HISEI of a family and the duration of early external childcare are negatively associated with problem behavior with peers.

**TABLE 5:** Bivariate correlations (two-tailed) of social competencies for children with a migration background.

Variable	1	2	3	4	5	6	7	8	9	10
1. Gender	1									
2. Siblings	–0.01	1								
3. CBQ: negative affect	–0.03	0.01	1							
4. CBQ: surgency	–0.10	0.10*	0.17**	1						
5. CBQ: effortful control	−0.27**	–0.04	−0.13*	0.04	1					
6. Home learning activities	0.19**	−0.18**	−0.11*	–0.01	−0.15**	1				
7. SES (HISEI)	0.02	−0.20**	–0.01	–0.08	−0.11*	0.20**	1			
8. Duration	0.01	−0.17**	0.01	−0.14**	–0.05	–0.02	0.27**	1		
9. SDQ: problem behavior with peers	–0.06	0.11*	0.19**	–0.10	0.08	–0.06	−0.15**	−0.19**	1	
10. SDQ: prosocial behavior	0.19**	0.07	−0.17**	0.06	−0.23**	0.13*	0.08	–0.02	−0.15**	1

N = 379; CBQ, Children’s Behavior Questionnaire; SES (HISEI), socioeconomic status; SDQ, Strength and Difficulties Questionnaire.*p < 0.05, **p < 0.01.

We included an overview of the correlations for the children without a migration background in order to compare them with the results of the children with a migration background. Table 6 provides the correlations between the social competencies, the duration of early external childcare, and the following control variables: gender; siblings; the CBQ scales negative affect, surgency, and effortful control; home learning activities; and HISEI. There is a correlation between problem behavior with peers and negative affect (CBQ), effortful control (CBQ), and HISEI (*r* = −0.06^∗^ to *r* = 0.16^∗∗^, ^∗^*p* < 0.05, ^∗∗^*p* < 0.01). Moreover, the variables gender, siblings, negative affect (CBQ), surgency (CBQ), effortful control (CBQ), and home learning activities correlate with prosocial behavior (*r* = −0.29^∗∗^ to *r* = 0.19^∗∗^, ^∗^*p* < 0.05, ^∗∗^*p* < 0.01). We also find a correlation between peer problems and prosocial behavior (*r* = −0.15, *p* < 0.01). The results of the correlation analysis differ from the results of children with a migration background. The following results remain the same: Girls are more likely to be assessed as inconspicuous in terms of their prosocial behavior as boys. Additionally, the temperament of a child matters in relation to both peer problems and prosocial behavior. Home learning activities are positively related to children’s prosocial behavior, and the HISEI of a family shows a negative effect on peer problems. The correlations in which the children without a migration background differ from the children with a migration background are the following: The number of siblings of children without a migration background show a positive effect on prosocial behavior. Further, the number of siblings and the duration of early external childcare is not correlated with peer problems in terms of children without a migration background.

**TABLE 6:** Bivariate correlations (two-tailed) of social competencies for children without a migration background.

Variable	1	2	3	4	5	6	7	8	9	10
1. Gender	1									
2. Siblings	0.01	1								
3. CBQ: negative affect	−0.09**	−0.07*	1							
4. CBQ: surgency	−0.16**	–0.04	0.27**	1						
5. CBQ: effortful control	−0.17**	–0.05	0.03	0.08**	1					
6. Home learning activities	0.15**	−0.13**	−0.07**	–0.04	−0.17**	1				
7. SES (HISEI)	–0.02	–0.04	–0.05	−0.12**	−0.07*	0.06*	1			
8. Duration	–0.07	−0.15**	0.02	0.02	0.07*	–0.04	0.08**	1		
9. SDQ: problem behavior with peers	–0.04	–0.03	0.16**	–0.02	0.08**	0.02	−0.06*	–0.02	1	
10. SDQ: prosocial behavior	0.19**	0.06*	−0.29**	−0.09**	−0.15**	0.17**	–0.02	–0.03	−0.15**	1

N = 1,226 CBQ, Children’s Behavior Questionnaire; SES (HISEI), socioeconomic status; SDQ, Strength and Difficulties Questionnaire.*p < 0.05, **p < 0.01.

### Group Differences in the Social Development of Children With a Migration Background Due to Different Durations of Early Institutional Childcare

The results regarding the effects of early institutional childcare on later social competencies of children with a migration background are presented in Table 7 (*N* = 379). The analysis reveals no main effect of childcare duration on prosocial behavior [*F*(2, 369) = 0.78, n.s., *f* = 0.06], but there is a main effect of childcare duration on problem behavior with peers [*F*(2, 369) = 6.19, *p* < 0.01, *f* = 0.18] (Table 7). The Bonferroni post-hoc estimated marginal means analysis reveals a significant difference (*p* < 0.01) between the peer problem scores of the childcare duration groups “up to 1 year” and “1–2 years” (0.57, 95% – CI [1.22, 1.02]), and between “up to 1 year” and “over 2 years” (0.59, 95% – CI [1.57, 1.03]). Two CBQ scales show an association between later problem behavior with peers {negative affect [*F*(1, 369) = 20.65, *p* < 0.001, *f* = 0.24] and surgency [*F*(1, 369) = 12.19, *p* < 0.001, *f* = 0.18]}. There are no group differences in later problem behavior with regard to the control variables gender, siblings, the CBQ scale effortful control, home learning activities, and HISEI (Table 7). For prosocial behavior, group differences are displayed for gender [*F*(1, 369) = 5.82, *p* < 0.05, *f* = 0.13], as well as all three CBQ scales: negative affect [*F*(1, 369) = 17.38, *p* < 0.001, *f* = 0.22], surgency [*F*(1, 369) = 5.47, *p* < 0.05, *f* = 0.12], and effortful control [*F*(1, 369) = 16.61, *p* < 0.001, *f* = 0.22]. Thus, there are no differences in prosocial behavior regarding siblings, home learning activities, and HISEI. The model for children with a migration background explains 10.5% of the variance for peer problems and 11.4% for prosocial behavior.

**TABLE 7:** Analysis of variance children with a migration background.

		Variable	*F* (*df*)	*p*-value	*f*
SDQ –problem behavior with peers	Main effect	Duration	6.19 (2)	<0.01	0.18
	Covariates	Gender	0.45 (1)	>0.05	0.04
		Siblings	2.81 (1)	>0.05	0.09
		CBQ: negative affect	20.65 (1)	<0.001	0.24
		CBQ: surgency	12.19 (1)	<0.001	0.18
		CBQ: Effortful control	2.66 (1)	>0.05	0.08
		Home learning activities	0.01 (1)	>0.05	0.00
		SES (HISEI)	3.41 (1)	>0.05	0.10
SDQ –prosocial behavior	Main effect	Duration	0.78 (2)	>0.05	0.06
	Covariates	Gender	5.82 (1)	<0.05	0.13
		Siblings	2.14 (1)	>0.05	0.08
		CBQ: negative affect	17.38 (1)	<0.001	0.22
		CBQ: surgency	5.47 (1)	<0.05	0.12
		CBQ: effortful control	16.61 (1)	<0.001	0.22
		Home learning activities	0.81 (1)	>0.05	0.04
		SES (HISEI)	1.57 (1)	>0.05	0.06

**N = 379; SDQ, Strength and Difficulties Questionnaire; CBQ, Children’s Behavior Questionnaire; SES (HISEI), socioeconomic status.**

In order to answer the research question of whether children with a migration background benefit in their social development from early external childcare, a second multivariate analysis of variance is performed, including all control variables. The results on later social competencies in the case of children from families *without a migration background* (*N* = 1,226) are presented in Table 8. The analysis shows neither a main effect of childcare duration on peer problems [*F*(2, 1,216) = 0.27, n.s., *f* = 0.00] nor on prosocial behavior [*F*(2, 1,216) = 0.18, n.s., *f* = 0.00] (Table 8). The results of the control variables show effects of all three CBQ scales {negative affect [*F*(1, 1,216) = 36.82, *p* < 0.001, *f* = 0.18], surgency [*F*(1, 1216) = 8.15, *p* < 0.01, *f* = 0.08], and effortful control [*F*(1, 1,216) = 7.65, *p* < 0.01, *f* = 0.08]} on later problem behavior with peers. Thus, the control variables gender, siblings, home learning activities, and HISEI do not show an association with later peer problems (Table 8). For prosocial behavior, group differences are displayed for the CBD scales negative affect [*F*(1, 1,216) = 93.57, *p* < 0.001, *f* = 0.29], and effortful control [*F*(1, 1,216) = 14.53, *p* < 0.001, *f* = 0.11]. Additionally, gender [*F*(1, 1,216) = 21.19, *p* < 0.001, *f* = 0.13] and home learning activities [*F*(1, 1,216) = 21.58, *p* < 0.001, *f* = 0.13] are related with later prosocial behavior. The control variables siblings, the CBD scale surgency, and HISEI do not show an association with later prosocial behavior. The model for children without a migration background explains 3.5% of the variance for problem behavior with peers and 13.5% for prosocial behavior.

**TABLE 8:** Analysis of variance children without a migration background.

		Variable	*F* (df)	*p*-value	*f*
SDQ –problem behavior with peers	Main effect	Duration	0.27 (2)	>0.05	0.00
	Covariates	Gender	1.46 (1)	>0.05	0.03
		Siblings	0.16 (1)	>0.05	0.00
		CBQ: negative affect	36.82 (1)	<0.001	0.18
		CBQ: surgency	8.15 (1)	<0.01	0.08
		CBQ: effortful control	7.65 (1)	<0.01	0.08
		Home learning activities	2.39 (1)	>0.05	0.45
		SES (HISEI)	3.79 (1)	>0.05	0.05
SDQ –prosocial behavior	Main effect	Duration	0.18 (2)	>0.05	0.00
	Covariates	Gender	21.19 (1)	<0.001	0.13
		Siblings	3.69 (1)	>0.05	0.05
		CBQ: negative affect	93.57 (1)	<0.001	0.29
		CBQ: surgency	0.34 (1)	>0.05	0.00
		CBQ: effortful control	14.53 (1)	<0.001	0.11
		Home learning activities	21.58 (1)	<0.001	0.13
		SES (HISEI)	1.86 (1)	>0.05	0.45

**N = 1,226; SDQ, Strength and Difficulties Questionnaire; CBQ, Children’s Behavior Questionnaire; SES (HISEI), socioeconomic status.**

## Discussion

This paper examines the association between the duration of early institutional childcare and the social development of children from families with a migration background. The correlations established here give a preliminary insight into the data. They show, initially, that relations do exist between the duration of early institutional childcare and problem behavior with peers. Whether there is not only a correlation, but also group differences when including relevant control variables depending on how long a child attends external childcare before the age of three, is shown by the results of the analysis of variance. A main effect of childcare duration (longer duration leading to less problem behavior with peers) was identified. Children with a background of migration exhibit fewer peer problems if they attend early institutional childcare for over 2 years or 1–2 years, compared to children who attended childcare for less than 1 year. In other words: The results indicate that children with a migration background who begin childcare at an earlier age differ in their social development from those who start later. A reason for the positive effect of childcare duration could be that children who attended early childcare for longer had more time to adapt to the new learning environment and its different rules and social conventions; consequently, they are capable of more positive interactions with their peers. Nevertheless, as the external care of children under 3 years of age is a relatively new phenomenon in Germany (with the exception of the former German Democratic Republic), there are some voices that are critical of early external childcare and, among other things, suggest it may even be harmful to children’s development (Neuß and Lorber, 2014). These assumptions are reconfirmed by international findings such as those of the NICHD Early Child Care Research Network (2003), which give indications of negative effects of early childcare. Note that those results mainly refer to a high intensity of early institutional childcare and a very early start of institutional childcare, which can hardly be found in Germany. However, as already mentioned, the results of international studies do not necessarily apply to Germany, since childcare systems differ substantially both in their organizational conception and with regard to patterns of demand and usage (International Labour Office, 2013; OECD, 2013). As such, our study does not confirm these negative effects; on the contrary, we find indications of positive effects for an early start to childcare. Our findings are in line with the results of other German studies like the NUBBEK study (Tietze et al., 2013) or the NEPS study conducted by Linberg et al. (2019). The fact that no negative effects of childcare duration were found in all three studies reinforces the importance of carrying out analyses within the framework of each system and of exercising caution regarding the transferability of international results. Although we use the same data as Linberg et al. (2019), the unique strength of this present study is that we focus on socially disadvantaged children; the results show that the effects are considerably stronger rather than weaker for these groups. To enable a more comprehensive understanding of the data, an analysis of variance for the children without a migration background, with the same control variables was performed. Nevertheless, the results do not show any significant main effects for children without a migration background. Even more important are the results for the children with a migration background: Our results show no negative effects of early external care for children with a migration background. There are several hypotheses to which the effect can be attributed. On the one hand there is a significant correlation between childcare duration and the SES of the family (*r* = 0.27), in the sense that children from families with a higher SES attend early external childcare for longer. This could mean that the effect that children who spend more time in early external childcare exhibit less problem behavior with peers could also be related to the SES of the family. On the other hand, early institutional childcare could be the reason for the positive effect on peer problems of children with a migration background at the age of 5 years. We conducted a robustness check excluding children from families with a high socioeconomic status in this subsample. The association between the duration of early external childcare during the first 3 years of a child’s life and problem behavior with peers remains significant, even within the group of children with lower SES [*F*(2, 182) = 6.02, *p* < 0.01, *f* = 0.27]—in fact, the effect strength actually increases. The same analysis including only children from high SES families with a migration background reveals a decreased effect of childcare duration on problem behavior with peers [*F*(2, 179) = 3.39, *p* < 0.05, *f* = 0.20]. Even though the effect decreases when only children from high SES families are included, it should not be over-interpreted since the effect is relatively small. In order to provide a complete statement about the differential effects of childcare duration for children from families with high or low SES with a migration background further research needs to be performed.

Although we find hints that children with a migration background benefit especially from an early start in institutional childcare, our results are not enough to determine a compensatory effect of early childcare. To claim such a compensatory effect further research needs to be implemented.

It is conceivable, for instance, that children with a migration background who exhibit more peer problems if they attend early institutional childcare for a shorter period do so because they may not yet have a sufficiently good knowledge of the German language; as a result, they may express themselves in a more physical way, which could be understood as problem behavior with peers. In order to check whether this assumption could be a possible explanation of the results, we conducted an additional analysis including vocabulary (in the German language) at the age of five in addition to all other control variables. The group difference for childcare duration in problem behavior with peers remains significant [*F*(2, 351) = 6.04, *p* < 0.01, *f* = 0.19]. Furthermore, vocabulary shows a significant effect with peer problems for children with a migration background [*F*(1, 351) = 6.22, *p* < 0.05, *f* = 0.13]. As the main effect of childcare duration is still present even when vocabulary is controlled for, this indicates that there is not only an effect mediated by language, but also an additional, direct effect of childcare duration. In some cases, pure institutional childcare may not be sufficient to reduce or even eliminate social and ethnical inequalities. At this point, the pedagogical quality of childcare must be emphasized, as the experiences children make in institutional childcare can be a direct result of its quality (Melhuish et al., 2015). Furthermore, the implementation of specific family-oriented measures that focus on parenting skills or the creation of stimulating learning environments, such as HIPPY or Opstapje, seems to be effective in supporting the parents, and thus the children (Schmidt, 2018).

The following limitations have to be addressed: In our study we include only the duration of childcare, but not the quality and intensity of early institutional childcare, as suggested by Kalb (2017). However, NEPS data does not provide sufficient measures of observed quality, and including intensity of care did not make any difference in the results (Linberg et al., 2019). One assumption of the missing effect of intensity is that children in Germany spend rather fewer hours in childcare compared to children from the USA (Linberg et al., 2019). In future analyses with other data, an integration of quality would be advisable in order to show whether this changes the effect of duration of early institutional childcare on children’s social development. It is important to note that there may have been misjudgments by parents who assessed the social development of their child too positively. This is indicated by the mean values, which are very low for problem behavior with peers and very high for prosocial behavior (Table 4); this could indicate processes of social desirability affecting answering patterns. This might also be an explanation for the low number of children with conspicuous SDQ scores (Table 3). The inconspicuousness of the SDQ scores in the sample must be taken into account when considering the significant differences in the levels of problem behavior with peers when controlling for the duration of early external childcare. Even if the children exhibit more peer problems, their scores often appear to still be in the normal range. Further, it must also be pointed out that the study’s implementation of the concept of migration background (at least one parent who has not acquired German as his/her first language) is rather broad and unspecific. The results may vary if the parent group “migrant background” were to be distinguished on the basis of both parents not speaking German as their first language, or the immigrant generation, or the type of migrant background (possibly by country or group of countries). The NEPS data shows that most parents with migration background enrolled in the study come from Russia, Poland, and Turkey. The sample sizes for other countries are considerably smaller, and therefore there is less empirical power to compare different immigrant groups. In order to establish whether the results change when including only children where both parents have not acquired German as their first language, we applied another robustness check. This analysis shows no association between duration and problem behavior with peers [*F*(2, 163) = 1.42, n.s., *f* = 0.13], which indicates that children with two parents having a migration background do not exhibit fewer peer problems when attending early external childcare sooner. This leaves us to question why children with at least one parent with a migration background differ in their problem behavior with peers when controlling for the duration of external childcare, but not children where both parents have a migration background. A descriptive investigation of the data showed that 37% of the group with two parents with a migration background attended institutional childcare (below the age of 3 years) for less than a year. In comparison, only 25.6% of the children with at least one parent with a migration background (this includes children with parents both having a migration background) and 12.4% of the children without a migration background attend external childcare below the age of 3 years for less than a year. The groups differ from one another and to fully answer the earlier addressed question further research is required. This leads us to another limitation of the used sample: 46.04% of the children with a migration background come from families with a high SES. Additionally, we find that the SES of the participants in the NEPS sample is higher than in the overall society (which is quite common when we look at longitudinal studies, which are based on the voluntariness of the participants), therefore our sample cannot be considered representative (Schimpl-Neimanns, 2004). This also results in a skewed distribution referring to duration in early institutional childcare in our sample: children from families with higher SES tend to start early external childcare earlier, meaning they attend for a longer duration. Based on the Statistisches Bundesamt (2019) one would expect a decrease in the number of children in each group as the duration increases, but actually the reported *n* (Table 4) increases. Still, the found effects remain the same but it is worth investigating whether they would increase if sampling issues were not prevalent.

Furthermore, the adjusted explained variance of the analysis of children with a migration background is rather low (peer problems 10.5%; prosocial behavior 11.4%). The small coefficients of determination show that the duration of early institutional childcare contributes little to the explanation of variance. Instead, individual differences between children, such as temperament, seem to correlate with social competencies. Thus, it is important when analyzing social competencies to consider not only children’s learning environments but also their individual characteristics (Bronfenbrenner and Morris, 2006). Potentially, the results would change if more children with conspicuous SDQ scores or more children from families with a low HISEI were included in the sample. This may be especially interesting when we look at the sample children without a migration background as the parents reported even less problem behavior with peers (*M* = 0.97 compared to *M* = 1.38 for children with a migration background).

This analysis—and the facts that, firstly, children with a migration background profit from an earlier start, but are underrepresented in childcare and that, secondly, when they do enter childcare, they do so at a later time—calls for a reduction in the barriers which impede access to early external childcare. This suggestion is reinforced by the work of Burghardt and Kluczniok (2016), which shows that parents with a migration background in particular assume childcare attendance to have a positive effect on their child’s development. In addition, Lokhande (2013) describes the access barriers specific to parents with a migration background. The insufficient number of early external childcare places available in Germany exacerbates the problem. In some cities, the child must be registered before or immediately after birth in order to get a chance of a place at an early childcare center. The results of our study have political and practical implications. Parents with a migration background are comparatively poorly informed about the early education system (Burghardt and Kluczniok, 2016), and are more likely to give “not receiving a childcare place” as a reason for non-use (Burghardt, 2019b). To reduce access barriers, both childcare providers and stakeholders could invest in information campaigns or provide written information in the parents’ native languages. As the parents have to register their child very early, the information on how to register could be provided by midwives or pediatricians, which would lead to an increased number of children with a migration background attending early external childcare.

## Conclusion

The present study aims at filling research gaps in Germany by focusing on the early years of children with a migration background with respect to their home and institutional learning environments. Overall, based on the analysis carried out, we find those children show small differences in their problem behavior with peers depending on the duration of early institutional childcare. Children with a migration background who attend institutional childcare for more than 1 year before the age of three show less problem behavior at the age of five than children who attended for less than 1 year. Our findings reinforce the debate to reduce barriers to access institutional childcare and to create equal educational opportunities for all children. The fact that children with a migration background and those with a low educational background are comparatively rarely cared for in early childhood institutions makes it clear that ethnic and social inequalities are already present at this young age, which can be understood as social disparities in the use of early childhood settings (Becker, 2012). Following Fuchs-Rechlin (2007), the course of processes of social inequality in the educational system is set before the beginning of the school career at an early biographical point in time. Knowing this and linking it to the results of the study calls for suppliers of external childcare and policy makers to ensure equal opportunities and combat social inequality. Increasing the informedness of this user group (as this is a basic requirement to access external childcare and can therefore be described as an access barrier) as discussed could lead to being able to put their care wishes into action.

Our results call for future research on the effects of early external childcare on disadvantaged children, especially as an overestimation of children’s competencies due to social desirability is plausible, and the differences may be greater than they appear in the present study.

## Data Availability Statement

The datasets presented in this study can be found in online repositories. The names of the repository/repositories and accession number(s) can be found below: Data from the National Educational Panel Study (NEPS): Starting Cohort Newborns, 10.5157/NEPS:SC1:6.0.0 which were used in this study are available as scientific use file. See http://www.neps-data.de.

## Ethics Statement

Ethical approval was not provided for this study on human participants because the data is from a scientific use file of the National Educational Panel, thus no additional ethical approval was obtained. It was not required in accordance with the national legislation and institutional requirements. Written informed consent to participate in this study was provided by the participants’ legal guardian/next of kin.

## Author Contributions

KK-R and LB: conceptualization and writing-review and editing. KK-R: organizing database and performing statistical analysis and writing-original draft preparation. All persons who meet authorship criteria are listed as authors, and both authors certify that they have participated sufficiently in the work. Both authors have read and agreed to the submitted version of the manuscript.

## Conflict of Interest

The authors declare that the research was conducted in the absence of any commercial or financial relationships that could be construed as a potential conflict of interest.

## Footnotes

1 In this paper, we will use the phrase ‘migration background’ to refer to those children who—regardless of ethnicity, immigrant generation, or current immigration status—have at least one parent who does not speak German as a first language at home.

2 In the beginning, NEPS data was collected as part of the Framework Program for the Promotion of Empirical Educational Research funded by the German Federal Ministry of Education and Research (BMBF). As of 2014, NEPS is carried out by the Leibniz Institute for Educational Trajectories (LIfBi) at the University of Bamberg in cooperation with a nationwide network.
