# Supplementary material for: Differences in Children’s Social Development: How Migration Background Impacts the Effect of Early Institutional Childcare Upon Children’s Prosocial Behavior and Peer Problems
Source: Front Psychol. 2021 Feb 16;12:614844. doi: 10.3389/fpsyg.2021.614844 (PMC7921488; doi:10.3389/fpsyg.2021.614844)
Supplement: Supplementary file 1 [file Table_1.DOCX]

**Supplementary Material**

| Table 1: *Model fits of the SDQ-scales* | | | | |
| --- | --- | --- | --- | --- |
| Model | χ^2^ | CFI | RMSEA | SRMR |
| one-factor model | 175.477 | .677 | .094 | .081 |
| two-factor model (problem) | 19.344 | .865 | .080 | .046 |
| two-factor model (prosocial) | 11.461 | .975 | .053 | .030 |
| *Note: N* = 454 | | | | |

| Table 2: *Mean values of the main variables of the initial and used sample* | | | | |
| --- | --- | --- | --- | --- |
|  | Initial sample | n | Used sample | n |
| Duration | 19.14 | 454 | 19.56 | 379 |
| (in months) |  |  |  |  |
| SDQ: Problem behavior with peers | 1.38 | 454 | 1.34 | 379 |
| SDQ: Prosocial behavior | 8.07 | 454 | 8.07 | 379 |

*Note:* The initial sample includes all children with a migration background and available SDQ values. The used sample excludes all children with missings in the control variables.
